# The Expected Behaviors of Posterior Predictive Tests and Their Unexpected Interpretation

**DOI:** 10.1093/molbev/msae051

**Published:** 2024-03-04

**Authors:** Luiza Guimarães Fabreti, Lyndon M Coghill, Robert C Thomson, Sebastian Höhna, Jeremy M Brown

**Affiliations:** GeoBio-Center, Ludwig-Maximilians-Universität München, Richard-Wagner-Str. 10, Munich 80333, Germany; Department of Earth and Environmental Sciences, Paleontology & Geobiology, Ludwig-Maximilians-Universität München, Richard-Wagner-Str. 10, Munich 80333, Germany; Center for Computation & Technology, Louisiana State University, Baton Rouge, LA 70803, USA; Present address: Division of Research, Innovation, and Impact & Department of Veterinary Pathobiology, University of Missouri, Columbia, MO 65211, USA; School of Life Sciences, University of Hawai‘i at Mānoa, Honolulu, HI 96822, USA; GeoBio-Center, Ludwig-Maximilians-Universität München, Richard-Wagner-Str. 10, Munich 80333, Germany; Department of Earth and Environmental Sciences, Paleontology & Geobiology, Ludwig-Maximilians-Universität München, Richard-Wagner-Str. 10, Munich 80333, Germany; Department of Biological Sciences and Museum of Natural Science, Louisiana State University, Baton Rouge, LA 70803, USA

**Keywords:** phylogenetics, Bayesian inference, model testing, RevBayes, posterior prediction

## Abstract

Poor fit between models of sequence or trait evolution and empirical data is known to cause biases and lead to spurious conclusions about evolutionary patterns and processes. Bayesian posterior prediction is a flexible and intuitive approach for detecting such cases of poor fit. However, the expected behavior of posterior predictive tests has never been characterized for evolutionary models, which is critical for their proper interpretation. Here, we show that the expected distribution of posterior predictive *P*-values is generally not uniform, in contrast to frequentist *P*-values used for hypothesis testing, and extreme posterior predictive *P*-values often provide more evidence of poor fit than typically appreciated. Posterior prediction assesses model adequacy under highly favorable circumstances, because the model is fitted to the data, which leads to expected distributions that are often concentrated around intermediate values. Nonuniform expected distributions of *P*-values do not pose a problem for the application of these tests, however, and posterior predictive *P*-values can be interpreted as the posterior probability that the fitted model would predict a dataset with a test statistic value as extreme as the value calculated from the observed data.

## Introduction

Statistical models are mathematical abstractions of reality that employ simplifying assumptions to capture important features of complex systems. As long as such assumptions do not depart from reality too strongly, statistical models can provide important insights into the systems they represent. However, if assumptions violate reality in meaningful ways, models lose both utility and reliability (Gelman et al. 2014; Brown and Thomson 2018).

Applied statistical fields, including phylogenetics and molecular evolution, need tools to assess when their models fail as meaningful abstractions of reality. The use of these tools is often referred to as testing absolute model fit or testing model adequacy. In a Bayesian framework, one way to test a model’s absolute fit is through posterior prediction (Rubin 1984; Bollback 2002).

Posterior prediction involves fitting a Bayesian model with parameters *θ* to observed data *y*. We then draw *S* values of *θ* from the posterior distribution, p(θ|y), and based on these posterior draws ( $\theta_{1}\cdots \theta_{S}$), we simulate *S* predictive datasets ( $y_{1}^{rep}\cdots y_{S}^{rep}$) of the same size as *y*. To perform a posterior predictive check of our model, we start by selecting a test statistic, T(y), that can be calculated on the observed and predictive datasets in order to compare them. One way to summarize the comparison between T(y) and $T(y_{1\cdots S}^{rep})$ is to calculate the fraction of predictive datasets that have test statistic values smaller or larger than the observed. If smaller, we can define the posterior predictive *P*-value as $Pr(T(y^{rep})<T(y)\;|\;y)$ and, if larger, $Pr(T(y^{rep})>T(y)\;|\;y)$ (see Höhna et al. 2018, for a description of different posterior predictive *P*-values). In either case, particularly large or small *P*-values indicate poor fit between the model and data.

The steps outlined above describe the mechanics of performing posterior prediction, but the more formal mathematical description of the quantity being estimated by this procedure is given by


(1) $$p=\int_{-\infty }^{T(y)}\left(\int_{-\infty }^{\infty }p(T(y^{rep})\;|\;\theta )p(\theta \;|\;y)\;d\theta \right)\;dT(y^{rep}).$$

Here, integration inside the parentheses describes the posterior predictive distribution of test statistic values, *T*, based on the posterior distribution of *θ*, while the outer integration describes calculation of the lower tail-area probability of this distribution with an upper limit defined by the empirical test statistic value, T(y).

Despite statistical literature discussing the behavior of posterior predictive tests in general (e.g. Meng 1994), expected distributions have never been characterized for posterior predictive *P*-values in phylogenetics and molecular evolution. Therefore, we aim to characterize the expected distributions of posterior predictive *P*-values for phylogenetics, compare such distributions across different types of test statistics, and understand how different parameters affect these expectations. To do so, we performed a broad set of simulations and posterior predictive analyses. We used the same model for simulation and analysis, and we drew parameter values for simulation from the prior distributions of the model parameters.

Our results convincingly demonstrate that posterior predictive *P*-values should not be interpreted like *P*-values from frequentist hypothesis tests. If misinterpreted in this way, posterior predictive tests will not be used to greatest effect and the strength of evidence for poor model fit will be underestimated because the expected distributions of posterior predictive *P*-values are, in many cases, highly nonuniform with a concentration of values near 0.5.

### Definition and Comparison of *P*-values

While posterior predictive *P*-values are called *P*-values because they involve the calculation of tail-area probabilities, they are distinct from several other types of *P*-values that we describe here for clarity.

The traditional frequentist *P*-value used in a hypothesis testing framework is defined as the probability of obtaining a test statistic value, $T(y^{rep})$ that is as or more extreme than the observed test statistic value, T(y), if the null hypothesis (with a value of *θ* fixed *a priori*) is true. If we focus on the probability of obtaining observations that are smaller than the observed, the frequentist hypothesis testing *P*-value can be described by the cumulative distribution function,


(2) $$p=\int_{-\infty }^{T(y)}p(T(y^{rep})\;|\;\theta )\;dT(y^{rep}).$$

Note that *θ*, and correspondingly the distribution of $T(y^{rep})$, does not depend at all on *y* in this case.

The parametric bootstrap *P*-value is similar in formulation to the frequentist *P*-value for testing a null hypothesis, but with estimated parameter $\hat{\theta }$. That is, instead of assuming a value of *θ* that is fixed *a priori*, we use the maximum-likelihood estimate, $\hat{\theta }$, based on *y*:


(3) $$p=\int_{-\infty }^{T(y)}p(T(y^{rep})\;|\;\hat{\theta })\;dT(y^{rep})$$

Parametric bootstrapping is a frequentist analog to posterior predictive model checking, but does not involve prior distributions or integration across different values of *θ*. The estimated value of $\hat{\theta }$ and the distribution of $T(y^{rep})$ do depend on *y* in this case.

The prior predictive *P*-value (Box 1980) is the Bayesian equivalent of the traditional frequentist hypothesis test, in the sense that the (probabilities of) parameter values defining the model are fixed *a priori* and do not depend on the observed data, *y*. The main difference is that, in the case of the prior predictive *P*-value, the cumulative distribution function is computed while integrating over different values of *θ* weighted by the *prior* probability of each, p(θ),


(4) $$p=\int_{-\infty }^{T(y)}\left(\int_{-\infty }^{\infty }p(T(y^{rep})\;|\;\theta )p(\theta )\;d\theta \right)\;dT(y^{rep}).$$

**Fig. 1.**
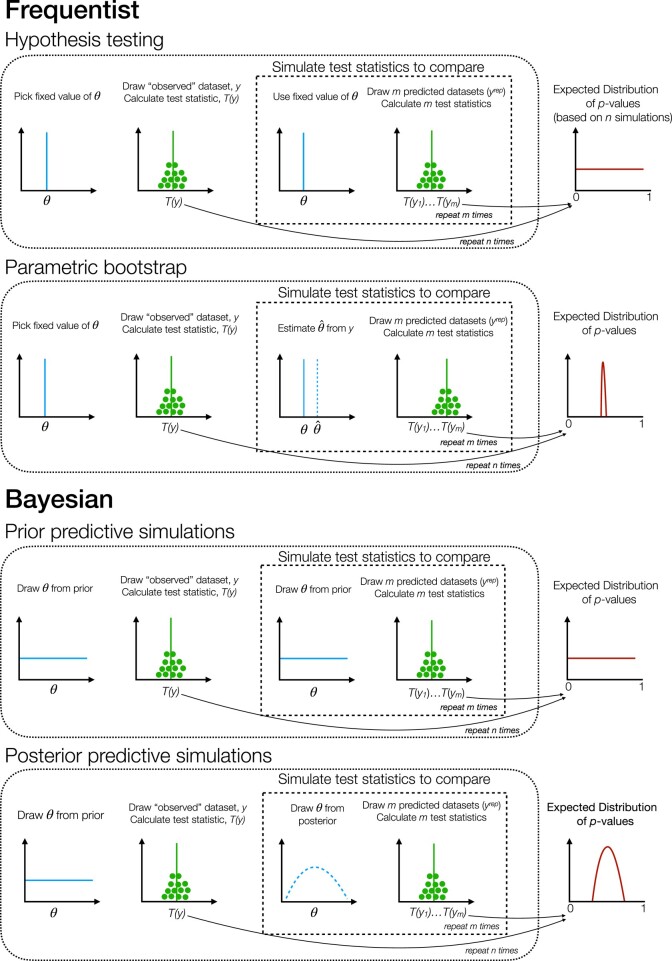
Schematic of workflows to estimate expected distributions for different types of *P*-values. The depictions of expected distributions are generalizations, intended to highlight important differences among different types of *P*-values.

A graphical depiction of the similarities and differences across *P*-values is given in Fig. 1.

## Results

**Fig. 2.**
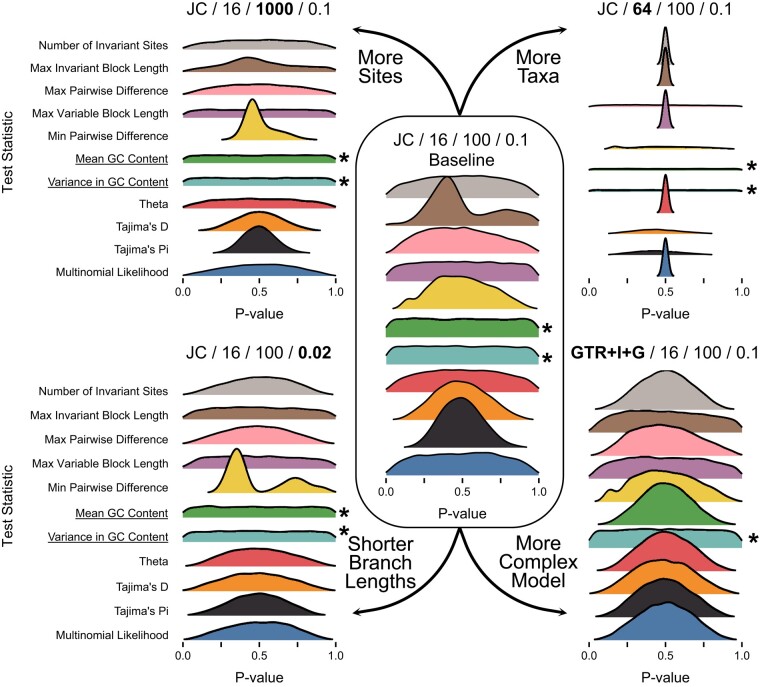
Distributions of posterior predictive *P*-values for data-based test statistics. The conditions for simulation and analysis are shown above each relevant portion of the figure as: Model/Number of Taxa/Number of Sites/Mean Branch Length. Results from the baseline setting (Setting 1 in Table 1) are shown in the middle. The other settings modified one condition of the baseline, indicated by the labels next to arrows. The Mean GC Content test statistic is ancillary for the JC model, while the Variance in GC Content test statistic is ancillary for both the JC and GTR+I+G models. The labels for these statistics are emphasized and the relevant distributions are marked with an asterisk.

**Fig. 3.**
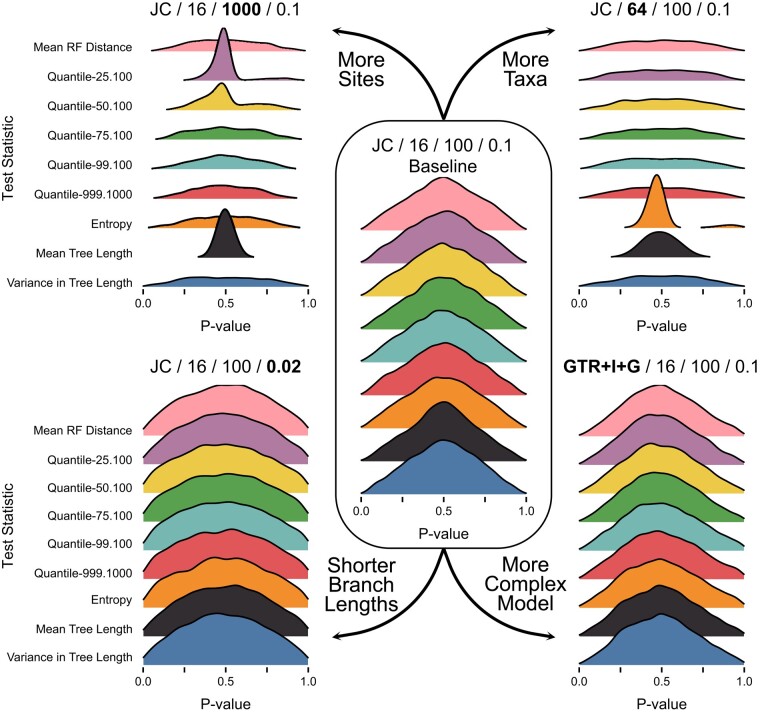
Distributions of posterior predictive *P*-values for inference-based test statistics. The labels and layout are the same as in Fig. 2.

The expected distribution of posterior predictive *P*-values varies by both test statistic and simulation condition, but is typically nonuniform (Figs. 2 and 3). Instead, these distributions are more concentrated around intermediate values, with fewer values near 0 or 1. This expectation has gone unappreciated in the discussion and applications of these tests to phylogenetics and molecular evolution (e.g. Bollback 2002; Brown 2014; Brown and Thomson 2018), but has important consequences for how results are interpreted.

In this study, we investigated the expected behavior of both data- and inference-based test statistics. Briefly, data-based test statistics can be calculated directly based on the properties of sequence alignments (e.g. the variance in GC content across sequences), while inference-based test statistics are calculated based on the properties of inferences conditional on those alignments and a model (e.g. the 99th percentile in the ordered vector of RF distances describing distances between trees sampled from the posterior distribution). Despite these differences, both types of test statistics have expected distributions that exhibit the same concentration of posterior predictive *P*-values near intermediate values.

While most test statistics have nonuniform expected distributions, ancillary test statistics (those statistics whose probability distributions do not depend on model parameters) should have uniform expected distributions (Meng 1994; Gelman 2013), because fitting the model has no effect for these statistics. This expectation explains the distributions of *P*-values for statistics based on GC content in our results (Fig. 2). Mean GC content is an ancillary statistic of the Jukes–Cantor model (JC), since this model assumes equal nucleotide frequencies, and we see roughly uniform expected distributions for Mean GC when using JC. However, Mean GC content is not ancillary for the GTR+I+G model, so the expected distribution is more concentrated around 0.5 in this case (bottom right of Fig. 2). Variance in GC content across sequences is ancillary for both models, since both assume that GC content does not vary across the tree. These distributions are roughly uniform for both models and far more dispersed than the distributions for other, nonancillary statistics.

Inference-based test statistics, by definition, depend on parameters of the model and cannot be ancillary. As a result, expected distributions of posterior predictive *P*-values for these statistics are never uniform (Fig. 3) and are always more concentrated near 0.5 than 0 or 1. Expected distributions for inference-based statistics tend to be more consistent than for data-based statistics, although some become markedly more peaked when dataset size increases either in terms of number of sites or number of taxa.

Several statistics, both data- and inference-based, have expected *P*-value distributions that are essentially fixed at 0.5 for some conditions (e.g. the effect of more taxa on the number of invariant sites or the topological entropy; Figs. 2 and 3). Posterior predictive *P*-values can be interpreted as the posterior probability of observing a test statistic value that is as extreme as the observed value (Gelman 2013), so these (nearly) invariant distributions may indicate that fitted models almost always predict datasets with (nearly) the same test statistic value as the observed. This interpretation makes sense for both the number of invariant sites and entropy test statistics with large numbers of taxa in our simulations (“Setting 2” in Table 1, “More Taxa” in Figs. 2 and 3). For datasets simulated with these conditions, nearly every site in the alignment will have some variation, causing the number of invariant sites to be approximately 0 for all observed and posterior predictive datasets. Similarly, these conditions lead to very diffuse posterior distributions of phylogenetic topologies, such that every topology sampled from the posterior distribution is unique and the estimated entropy is the same across datasets.

**Table 1:** Settings for simulations and posterior predictive analyses

Setting	Substitution Model	Number of taxa	Number of sites	Mean branch length
1 (Baseline)	JC	16	100	0.1
2	JC	64	100	0.1
3	JC	16	1,000	0.1
4	JC	16	100	0.02
5	GTR +Γ+ I	16	100	0.1

Expected *P*-value distributions for some test statistics are multimodal (e.g. the minimum pairwise difference statistic; Fig. 2). Multimodal distributions typically occur with discrete test statistics that adopt a small number of possible values. Such distributions are not unique to phylogenetics and molecular evolution and present no particular difficulties for interpretation (Gelman 2013). However, these expected distributions are worth bearing in mind when interpreting such values in empirical studies. In these cases, small changes in test statistic values can lead to seemingly large changes in *P*-values.

**Fig. 4.**
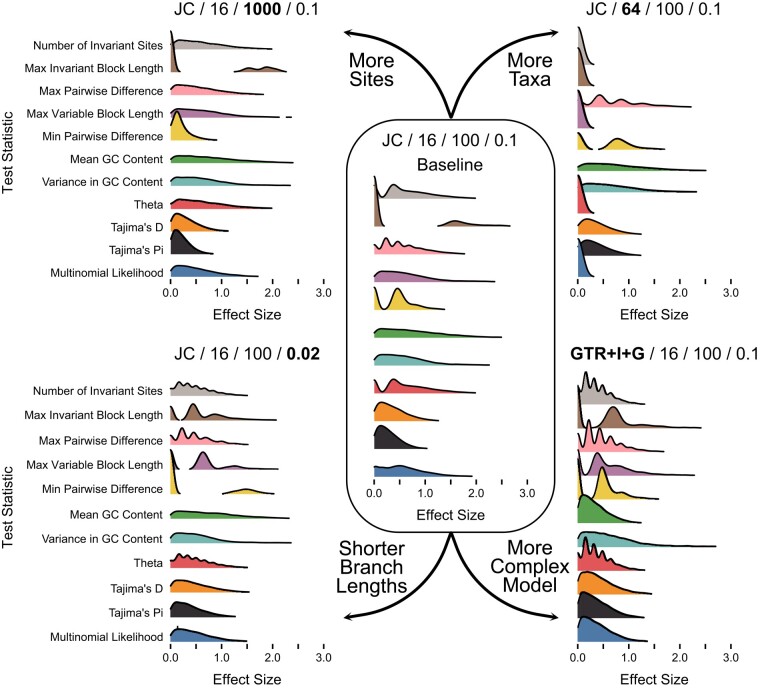
Distributions of effect sizes for data-based test statistics. The labels and layout are the same as in Fig. 2.

**Fig. 5.**
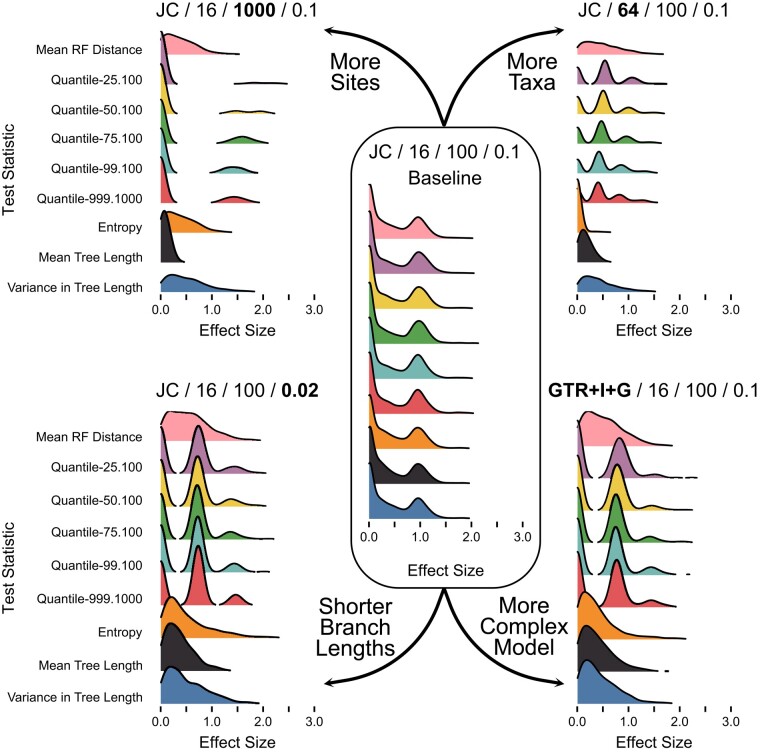
Distributions of effect sizes for inference-based test statistics. The labels and layout are the same as in Fig. 2.

While *P*-values have received the most attention as a way to summarize the results of posterior predictive tests, an alternative approach is the use of effect sizes (Doyle et al. 2015; Höhna et al. 2018). Briefly, effect sizes measure the distance between the empirical test statistic value and the median of the posterior predictive distribution, normalized by the standard deviation of the posterior predictive distribution. Effect sizes are useful for understanding the magnitude of the discrepancy between the observed and predicted values, even when the observed value is highly improbable given the model. We used the same set of simulations and analyses to characterize the expected distributions of effect sizes (Figs. 4 and 5). These expected effect size distributions make sense in light of the expected distributions of *P*-values, although there is a preponderance of values near 0 rather than near 0.5. Due to the way effect sizes are calculated, their expected distribution is not uniform even when the expected distribution of *P*-values is uniform. As an example, see the distributions of expected effect sizes for Mean GC content for any of the analyses employing a JC model (Fig. 4). As with expected distributions of *P*-values, many of the distributions of effect sizes are multimodal, due to the discrete nature of many test statistics. However, in all cases, these values are nearly always < 2.0. This result stands in contrast to our experiences analyzing empirical data sets, where effect sizes are frequently ≫ 10.0 (Doyle et al. 2015).

## Discussion and Conclusions

*P*-values by definition represent the probability, conditional on the model, of observing data that are more extreme than what has actually been observed. A *P*-value that is very small or very large indicates that the observed dataset is an outlier relative to model expectations and possibly reflects poor absolute model fit. In a standard frequentist hypothesis test, the model corresponds to the null hypothesis and poor model fit would lead to its rejection. Frequentist *P*-values for hypothesis testing are explicitly constructed to have uniform distributions in order to control false positive rates. Importantly, this uniformity of *P*-values stems from the use of fixed (i.e. not fitted) parameter values.

Posterior predictive *P*-values, on the other hand, use model parameter values that have been fitted to the observed data (Fig. 1). The probability that the observed data are more extreme than expected is always reduced relative to tests using fixed values, because the model is given the opportunity to explain the data as well as possible. Thus, expected distributions of posterior predictive *P*-values tend to be concentrated around 0.5 (Meng 1994, Figs. 2 and 3), although the precise shape can vary by both test statistic and analysis condition. In practice, nonuniform distributions can be precisely what we want if our goal is to assess the ability of our model to capture certain aspects of our observed data (Gelman 2013). If our model always does a good job of predicting these features, then the expected distribution of posterior predictive *P*-values should reflect that.

We focused here on posterior predictive *P*-values, computed in a Bayesian framework, but similar considerations apply to *P*-values from parametric bootstrapping analyses (Fig. 1) conducted in a frequentist framework. Since parametric bootstrapping also involves fitting a model to a dataset, it should also produce expected distributions of *P*-values that are nonuniform. In fact, expected distributions from parametric bootstrapping may be much more concentrated than those from posterior prediction, because the effect of posterior uncertainty keeps the expected distributions from becoming too peaked in a Bayesian setting.

If posterior predictive *P*-values are misinterpreted as frequentist hypothesis testing *P*-values, the evidence for poor model fit will usually be underestimated. A posterior predictive *P*-value of 0.05 typically has a <5% probability of occurring when the assumptions of an analysis exactly match the data-generating process. However, again, it is best to avoid framing posterior predictive tests in frequentist hypothesis testing terms. The goal of posterior predictive tests should not be to reject a model as “true” (Gelman et al. 2014), since we know that none of the models fully represent the complexity of real evolutionary processes. Rather, these tests indicate the extent to which the model’s simplifications are problematic for explaining important features of the data.

In the course of this study, we simultaneously characterized expected distributions of posterior predictive *P*-values for multiple test statistics and our results demonstrate that many of these test statistics are correlated. Strong correlations mean that a count of the number of statistics with small *P*-values is not an effective way to measure the overall degree of fit between model and data. Small *P*-values for posterior predictive tests with two uncorrelated test statistics would provide more insight than small *P*-values for many such tests with highly correlated test statistics.

Empirical application of posterior predictive tests in phylogenetics and molecular evolution has frequently resulted in extremely small *P*-values across many different datasets using a variety of different test statistics (e.g. Foster 2004; Lartillot et al. 2007; Zhou et al. 2010; Doyle et al. 2015; Duchêne et al. 2016; Höhna et al. 2018; Richards et al. 2018). Based on the nature of the expected distributions that we have characterized here, these empirical results often represent even stronger evidence than has been appreciated that commonly applied models in phylogenetics are seriously inadequate. Depending on which particular test statistics exhibit poor fit for a given study, we may take different actions depending on the goals of the analysis. For instance, previous work has shown that inadequately accounting for variation in substitution rates across sites can have detrimental consequences on phylogenetic accuracy (Yang 1996), and so poor fit for the number of invariant sites test statistic might motivate exploration of alternative types of distributions or models for characterizing rate variation. Poor fit for other test statistics, for instance the max invariant block length, may have less of an impact on phylogenetic accuracy, but still indicate interesting patterns of molecular evolution that are worth exploring further (e.g. highlighting striking spatial patterns of constraint, like those found in ultraconserved elements).

An important future direction for this work will be to more comprehensively characterize the aspects of empirical datasets that consistently exhibit poor fit under commonly employed models of sequence and trait evolution. These results would highlight which aspects of the molecular evolutionary process are most likely to lead to analytical issues and that are least well captured by available models, thereby helping to prioritize and guide the efficient development of more effective new models. Such information would also help identify interesting and widespread patterns of genomic evolution still in need of explanation.

To our knowledge, this paper is the first characterization of the expected distribution of posterior predictive *P*-values for models commonly used in phylogenetics and molecular evolution. Our hope is that the results presented here clarify the interpretation of empirical assessments of absolute model fit using posterior predictive tests. These tests can highlight important mismatches between model assumptions and the actual biological processes that shape genome sequences. Critical thought must be given to how models are applied in order to gain insight into evolutionary patterns and processes (Brown and Thomson 2018).

## Materials and Methods

### Data Simulation

To understand the expected distributions of posterior predictive *P*-values when analysis conditions precisely match those under which the data were generated, we first simulated alignments of DNA sequences using a baseline set of conditions: a JC model (Jukes and Cantor 1969) of sequence evolution, a 16-taxon tree from a uniform distribution, alignments with 100 sites, and exponentially distributed branch lengths with a mean of 0.1 (Table 1, Setting 1). We then simulated alignments under four additional sets of conditions that varied each baseline setting individually. We increased the size of the tree to 64 taxa (Setting 2), increased the length of the alignment to 1,000 sites (Setting 3), reduced the mean branch length to 0.02 (Setting 4), and used the General Time-Reversible model (GTR) (Tavaré 1986) with Gamma-distributed rate variation among sites as four discrete rate categories (*Γ*) (Yang 1994, 1996) and a proportion of invariable sites (I) (Adachi and Hasegawa 1995; Gu et al. 1995) (Setting 5). For each setting, we simulated 10,000 alignments in RevBayes (Höhna et al. 2016) by randomly drawing parameter values from the prior distribution associated with each parameter (see Table 2 for details about the parameters and their prior distributions). Parameter values were drawn separately for each dataset.

**Table 2:** Parameters of phylogenetic models and their associated prior distributions

Parameter	Description	Prior distribution	Parameters of the distribution
*Ψ*	Topology of the tree	Uniform	Num. of Taxa=16 or 64
bl	Branch lengths	Exponential	$\lambda_{bl}=10$ or 50
*π*	Equilibrium base frequencies	Dirichlet	$\alpha_{\pi }=(1,\;1,\;1,\;1)$
er	Exchangeabilities	Dirichlet	$\alpha_{er}=(1,\;1,\;1,\;1,\;1,\;1)$
*α*	Shape of the Gamma distribution	Exponential	$\lambda_{\alpha }=0.05$
I	Proportion of invariant sites	Beta	$\left(\alpha_{I},\;\beta_{I})=(10,\;20\right)$

Once datasets were simulated, we conducted Bayesian Markov chain Monte Carlo (MCMC) analyses using RevBayes (Höhna et al. 2016) to estimate posterior distributions of tree topologies and model parameter values for each simulated dataset. We then drew samples from these posterior distributions to generate posterior predictive datasets and compared each original dataset to its corresponding posterior predictive distribution using a variety of test statistics (Höhna et al. 2018). Details of these analyses are provided below.

### Markov Chain Monte Carlo Analyses

We performed MCMC analyses in RevBayes (Höhna et al. 2016) for each simulated dataset using the same conditions under which they were simulated (see Table 1). Prior distributions were the same as those from which parameter values were drawn for simulation (Table 2). For all analyses, we estimated the tree topology and branch lengths. For analyses of datasets simulated under Setting 5 with a GTR+*Γ*+I model, we also estimated the equilibrium base frequencies, the exchangeabilities, the shape parameter of the *Γ* distribution, and the proportion of invariable sites (I; Table 2). Each analysis involved a burn-in phase of 200 iterations, followed by MCMC sampling for 10,000 iterations. The moves used for each parameter, and their associated weights, are provided in Table 3. A subset of runs from different conditions were spot checked to ensure that the MCMC settings were sufficient to achieve good convergence of both scalar parameter values and tree topologies. MCMC analyses conducted for use with posterior predictive analyses involving data-based test statistics used two independent replicate analyses and automatic tuning of moves every 200 generations during both the burn-in and sampling phases. Analyses conducted for use with posterior predictive analyses involving inference-based test statistics used a single replicate and only used automatic tuning during the burn-in phase.

**Table 3:** Moves used during Markov chain Monte Carlo (MCMC) analyses

Function in RevBayes	Description	Parameter to change	Weight
mvNNI	Nearest-neighbor interchange move	*Ψ*	num. of taxa (e.g. 16, 64)
mvSPR	Subtree prune-and-regraft move	*Ψ*	num. of taxa x 0.1 (e.g. 1.6, 6.4)
mvBranchLengthScale	Scaling move on the branch lengths	bl	num. of taxa (e.g. 16, 64)
mvBetaSimplex	Scaling move on nucleotide frequencies	*π*	2.0
mvDirichletSimplex	Scaling move on nucleotide frequencies	*π*	1.0
mvBetaSimplex	Scaling move on exchangeabilities	er	3.0
mvDirichletSimplex	Scaling move on exchangeabilities	er	1.5
mvScale	Scaling move on shape parameter	*α*	2.0
mvBetaProbability	Scaling move on proportion of invariable sites	I	2.0

JC analyses used only the first three moves

### Posterior Predictive Analyses and *P*-values

To perform posterior predictive analysis on each of the simulated datasets, we used the P^3^ (Phylogenetic Posterior Prediction) workflow implemented in RevBayes (Höhna et al. 2018). Phylogenetic posterior predictive analyses involve four steps: (1) estimating posterior distributions of phylogenetic trees and model parameters from input data (see above), (2) simulating new (posterior predictive) data using parameter values drawn from the estimated posterior distributions, (3) computing test statistics for both the original and simulated data, and (4) calculating *P*-values and effect sizes to summarize the (dis)similarity between original and simulated data. Some test statistics, known as inference-based (see below), may depend on characteristics of the inferences drawn from data. To calculate these, an additional step (3a) is necessary that involves running MCMC analyses on each simulated, posterior predictive dataset. For step (2), we simulated 1,001 posterior predictive datasets when using data-based test statistics and 501 posterior predictive datasets when using inference-based test statistics.

The P^3^ workflow has a number of test statistics (Tables 4 and 5) available that summarize characteristics of alignments. Some of these statistics (data-based, Table 4) are calculated directly from the alignment itself, while others (inference-based, Table 5) are calculated based on characteristics of inferences drawn from the alignment. We used all test statistics currently implemented in P^3^ in RevBayes. For any of these statistics, *P*-values can be used to assess whether the “observed” alignment is similar to the posterior predictive alignments (Doyle et al. 2015; Höhna et al. 2018). *P*-values indicate what percentage of posterior predictive test statistic values are more extreme than the observed value.

**Table 4:** Descriptions of data-based test statistics

Test Statistic	Description	Reference
Number of invariant sites	Number of columns in the alignment that show no variation in nucleotide content	Höhna et al. (2018)
Max invariant block length	The maximum number of consecutive sites with no variation	
Max pairwise difference	The scaled number of mismatches between the pair of sequences with the greatest number of mismatches	Höhna et al. (2018)
Max variable block length	The maximum number of consecutive sites with variation	
Min pairwise difference	The scaled number of mismatches between the pair of sequences with the fewest number of mismatches	Höhna et al. (2018)
Mean GC content	GC content averaged across all sequences	Höhna et al. (2018)
Variance in GC content	Variance in GC content across sequences in an alignment	Höhna et al. (2018)
Theta	Watterson’s *θ* measures the genetic diversity in a given population	Watterson (1975)
Tajima’s D	Accounts for how much the variability observed is due to chance	Tajima (1989)
Tajima’s *π*	Average number of pairwise differences across sequences in an alignment	Nielsen and Slatkin (2013) and King et al. (2018)
Multinomial likelihood	Measures the ability of the model to account for different site pattern frequencies	Goldman (1993)

**Table 5:** Descriptions of inference-based test statistics, originally described by Brown (2014)

Test Statistic	Description
Mean RF	Mean RF (Robinson and Foulds (1981)) distance between trees sampled from the posterior distribution
Quant 25	25th percentile in the ordered vector of RF distances between trees sampled from the posterior distribution
Quant 50	50th percentile in the ordered vector of RF distances between trees sampled from the posterior distribution
Quant 75	75th percentile in the ordered vector of RF distances between trees sampled from the posterior distribution
Quant 99	99th percentile in the ordered vector of RF distances between trees sampled from the posterior distribution
Quant 999	999th 1,000-quantile in the ordered vector of RF distances between trees sampled from the posterior distribution
Entropy	Gain in information about the tree topology provided by the data
Mean TL	Mean length of trees sampled from the posterior distribution
Var TL	Variance in the length of trees sampled from the posterior distribution

*P*-values near 0 or 1 indicate that the observed value falls in a tail of the posterior predictive distribution. Midpoint *P*-values are particularly useful for discrete test statistics, where ties can be observed between posterior predictive and observed values. In such a case, the midpoint *P*-value will consider half of the tied posterior predictive values to be more extreme than observed and half to be less extreme than observed. In this study, we specifically focused on the lower, one-tailed, midpoint *P*-value. All 10,000 simulated datasets were analyzed to characterize the behavior of posterior predictive analyses for data-based test statistics, while 1,000 datasets were analyzed for inference-based test statistics due to their more computationally intensive calculation.

### Effect Sizes

While we have largely focused our attention in this study on the distribution of posterior predictive *P*-values, because such values have received the most attention in the statistical literature, an alternative measure of absolute model fit is the posterior predictive effect size (PPES; Doyle et al. 2015; Höhna et al. 2018). Complementary to posterior predictive *P*-values, posterior predictive effect sizes capture the magnitude of differences between observed and expected test statistic values on a broader scale. While posterior predictive tests using two different test statistics for the same dataset may both produce *P*-values of 0, one observed value may fall just outside the tails of the corresponding posterior predictive distribution, while the other observed value may be very, very far away from its predicted values. Effect sizes differentiate between these two situations, and are calculated as


(5) $$PPES=\frac{\left|T(y)-M(p(T(y^{rep})\;|\;y))\right|}{\sigma (p(T(y^{rep})\;|\;y))}$$

where *y* is the observed dataset, $y^{rep}$ is a posterior predictive dataset, T(y) is a test statistic calculated with *y*, $p(T(y^{rep})\;|\;y)$ is the posterior predictive distribution of *T*, *M* is the median of a distribution, and *σ* is the standard deviation of a distribution. In other words, a posterior predictive effect size is the absolute value of the difference between the observed test statistic value and the median of the posterior predictive distribution of test statistic values, normalized by the posterior predictive distribution’s standard deviation. Using the same simulations and analyses that we used to understand the expected behavior of posterior predictive *P*-values, we also examined the expected distributions of posterior predictive effect sizes.

## Data Availability

No new data were generated or analyzed in support of this research.
